# *Tm*IKKε Is Required to Confer Protection Against Gram-Negative Bacteria, *E. coli* by the Regulation of Antimicrobial Peptide Production in the *Tenebrio molitor* Fat Body

**DOI:** 10.3389/fphys.2021.758862

**Published:** 2022-01-07

**Authors:** Hye Jin Ko, Bharat Bhusan Patnaik, Ki Beom Park, Chang Eun Kim, Snigdha Baliarsingh, Ho Am Jang, Yong Seok Lee, Yeon Soo Han, Yong Hun Jo

**Affiliations:** ^1^Department of Applied Biology, Institute of Environmentally-Friendly Agriculture, College of Agriculture and Life Sciences, Chonnam National University, Gwangju, South Korea; ^2^Department of Biosciences and Biotechnology, Fakir Mohan University, Balasore, India; ^3^Department of Biology, College of Natural Sciences, Soonchunhyang University, Asan, South Korea

**Keywords:** *Tenebrio molitor*, IMD pathway, IKKε, antimicrobial peptides, RNAi

## Abstract

The inhibitor of nuclear factor-kappa B (NF-κB) kinase (IKK) is the core regulator of the NF-κB pathway against pathogenic invasion in vertebrates or invertebrates. IKKβ, -ε and -γ have pivotal roles in the Toll and immune deficiency (IMD) pathways. In this study, a homolog of IKKε (*Tm*IKKε) was identified from *Tenebrio molitor* RNA sequence database and functionally characterized for its role in regulating immune signaling pathways in insects. The *TmIKKε* gene is characterized by two exons and one intron comprising an open reading frame (ORF) of 2,196 bp that putatively encodes a polypeptide of 731 amino acid residues. *Tm*IKKε contains a serine/threonine protein kinases catalytic domain. Phylogenetic analysis established the close homology of *Tm*IKKε to *Tribolium castaneum* IKKε (*Tc*IKKε) and its proximity with other IKK-related kinases. The expression of *TmIKKε* mRNA was elevated in the gut, integument, and hemocytes of the last-instar larva and the fat body, Malpighian tubules, and testis of 5-day-old adults. *TmIKKε* expression was significantly induced by *Escherichia coli*, *Staphylococcus aureus*, and *Candida albicans* challenge in whole larvae and tissues, such as hemocytes, gut, and fat body. The knockdown of the *TmIKKε* messenger RNA (mRNA) expression significantly reduced the survival of the larvae against microbial challenges. Further, we investigated the induction patterns of 14 *T. molitor* antimicrobial peptides (AMPs) genes in *TmIKKε* gene-silencing model after microbial challenges. While in hemocytes, the transcriptional regulation of most AMPs was negatively regulated in the gut and fat body tissue of *T. molitor*, AMPs, such as *TmTenecin 1*, *TmTenecin 4*, *TmDefensin*, *TmColeoptericin A*, *TmColeoptericin B*, *TmAttacin 1a*, and *TmAttacin 2*, were positively regulated in *TmIKKε*-silenced individuals after microbial challenge. Collectively, the results implicate *TmIKKε* as an important factor in antimicrobial innate immune responses in *T. molitor*.

## Introduction

The innate immune response represents the first line of defense in vertebrates and it is the only defense arsenal in invertebrates against microbial infections (Hoffmann et al., 1999). This is because of a lack of adaptive immune strategy in invertebrates that necessitates the over-reliance on the innate immune cascades for defense against microbial infection (Hoffmann et al., 1999; Li and Xiang, 2013). Antimicrobial peptides (AMPs) production represents one of the crucial effector mechanisms of innate immunity in insects. AMPs attribute insects most resistant to bacterial infections and shed applications as novel microbicides (Wu et al., 2018). Since the discovery of the first insect AMP called cecropin from the giant silk moth *Hyalophora cecropia* (Hultmark et al., 1980), over 150 AMPs have been identified, purified, and characterized from insects. Drosomycin, an antifungal peptide, and diptericin, an antibacterial peptide, have been identified and characterized in *Drosophila melanogaster* (Lemaitre et al., 1996; Nicolas et al., 1996). Attacin B, which is one of two attacin genes identified from *Hyphantria cunea*, is strongly induced by gram-positive and gram-negative bacteria (Kwon et al., 2008). Another attacin gene identified from *Spodoptera exigua* has antimicrobial activity against *Escherichia coli* DH5α strain, *Pseudomonas cichorii*, *Bacillus subtilis*, and *Listeria monocytogenes* (Bang et al., 2012). In the *Tenebrio* model, *in silico* analysis and induction patterns of AMP genes that include tenecin-1 (defensin family), -2 (coleoptericin family), -3 (thaumatin-like protein family), and -4 (attacin family), thaumatin-like protein (TLP)-1, and -2, Attacin-1a, -1b, and -2, Defensin and Defensin-like, Coleoptericin-A, -B, and -C, and Cecropin-2 have been studied (Kim et al., 1998, 2017; Roh et al., 2009; Chae et al., 2012; Johnston et al., 2013; Zhu et al., 2014; Noh and Jo, 2016; Jo et al., 2018; Maistrou et al., 2018; Jang et al., 2020a,b; Ali Mohammadie Kojour et al., 2021).

In insects, AMPs are induced through the activation of two key signaling cascade mechanisms – the Toll and immune deficiency (IMD) pathways. In *Drosophila*, the IκB kinase (*Dm*IKK) complex, which is the major component of the IMD pathway, stimulates the activation of the nuclear factor-kappa B (NF-κB) protein Relish by phosphorylation (Georgel et al., 2001; Vidal et al., 2001; Chen et al., 2002; Choe et al., 2002, 2005; Gottar et al., 2002; Ramet et al., 2002; Cha et al., 2003; Leulier et al., 2003; Silverman et al., 2003; Kaneko et al., 2004). In addition, lipopolysaccharide (LPS) and peptidoglycan (PGN) from gram-negative bacteria stimulate the IMD pathway in *Drosophila* (Leulier et al., 2003; Kaneko et al., 2004). Further, IKKε in *D. melanogaster* not only plays a principal role in IMD regulation but also phosphorylates DIAP1 and controls janus kinase (JNK) activation and apoptosis (downstream of IMD) (Gan et al., 2021). This study demonstrated that the activation of the IMD pathway against sindbis virus (SINV) infection is highly dependent on the microbiota present in the gut (GT) of *Aedes aegypti* (Barletta et al., 2017). In another insect model, *Bombyx mori*, the expression of CecropinA1 is regulated by Relish in response to gram-negative bacteria (Hua et al., 2016). In addition, the direct functions of *B. mori* peptidoglycan recognition protein L1 (*Bm*PGRP-L1) and *Bm*IMD in the IMD pathway are suspected but not clearly identified (Zhan et al., 2018). In *Plutella xylostella*, IMD RNA interference (RNAi) affected the expression of the downstream genes of the IMD pathway (Lin et al., 2018). In fact, several studies have characterized the Toll and IMD pathway responses of diverse insects including *Plautia stali* stink bugs (Nishide et al., 2019), aphids (International Aphid Genomics Consortium, 2010), kissing bugs (Mesquita et al., 2015; Salcedo-Porras and Lowenberger, 2019), or other arthropods such as *Tetranychus* mites (Palmer and Jiggins, 2015; Santos-Matos et al., 2017) and shrimp (Li et al., 2019). Additionally, the crosstalk between Toll and IMD pathways in stink bugs by the RNAi experiments of Toll and IMD pathways-related genes, including *PsImd*, *PsMyD88*, *PsDorsal*, *PsPGRP-L1a*, *PsPGRP-L1b*, *PsPGRP-L2*, *PsLysM*, *PsGNBP1* have been suggested (Nishide et al., 2019).

In the beetle *Tribolium castaneum*, the PGRP-LA may be a pivotal sensor of the IMD pathway for both gram-negative and gram-positive bacteria, and both PGRP-LC and -LE acts as IMD pathway-associated sensors, mainly for gram-negative bacteria (Koyama et al., 2015). Based on comparative genomic analysis, around 300 candidate defense proteins were identified and clustered depending on the immune pathway such as Toll, IMD, and JAK-STAT pathways (Zou et al., 2007). Inducible immune-related genes, including Toll, PGRP, and AMP genes such as ferritin, defensin, and others against crude LPS were identified using the suppression subtractive hybridization (SSH) method, and antifungal activity of recombinant TLP was assayed in *T. castaneum* (Altincicek et al., 2008).

In contrast, a comprehensive study of the IMD pathway in innate immune responses against infections by various pathogens has been partially performed in *T. molitor* with the functional characterization of *Tm*PGRP-LE, *Tm*IMD, *Tm*IKKγ, and *Tm*Relish (Tindwa et al., 2013; Jo et al., 2019; Ali Mohammadie Kojour et al., 2020; Keshavarz et al., 2020b,c,d; Ko et al., 2020). In this study, we identified the IκB kinase ε (IKKε) gene, one of the important components for the IMD pathway, from *T. molitor* RNA and DNA sequence database. We investigated the mRNA expression patterns of *Tm*IKKε depending on different developmental stages, tissues, and microbial challenges to the host. Moreover, we investigated the effects of *TmIKKε-*specific knockdown on larval mortality, AMP production, and expression of NF-κB genes against various pathogens. The findings that *IKKε* knockdown beetles are especially susceptible to *E. coli* but not a gram-positive bacterium or fungus parallels a recent study wherein fruit flies lacking their major AMP genes were specifically susceptible to gram-negative bacteria, but not so much gram-positive bacteria or fungi (Hanson et al., 2019). Collectively, our data provide a better understanding of the IMD pathway in the *Tenebrio* innate immune response.

## Materials and Methods

### Insect Rearing

*Tenebrio molitor* larvae were reared in the dark at 27°C ± 1°C and 60% ± 5% relative humidity in an environmental chamber established in the laboratory. The reared larvae were fed an artificial diet (170 g wheat flour, 20 g roasted soy flour, 10 g protein, 100 g wheat bran, 0.5 g sorbic acid, 0.5 mL propionic acid, and 0.5 g chloramphenicol in 200 mL of distilled water, sterilized by autoclaving at 121°C for 15 min). Healthy 10th to 12th instar larvae (1.2–1.5 cm length) were used for experiments.

### Preparation of Microorganisms

Three microorganisms—the gram-negative bacterium *E. coli* strain K12, gram-positive bacterium *S. aureus* strain RN4220, and the fungus *C. albicans*—were used for immune challenge experiments. *E. coli* and *S. aureus* were cultivated in Luria-Bertani (LB) broth (MB cell, South Korea). *C. albicans* suspension was prepared by culturing the fungi in Sabouraud dextrose broth (MB cell) at 37°C overnight. The microorganisms were harvested and washed twice in 1× phosphate-buffered saline (PBS) (8.0 g NaCl, 0.2 g KCl, 1.42 g Na_2_HPO_4_, 0.24 g of KH_2_PO_4_ in 1 L of distilled water, pH 7.0) and centrifuged at 1,700 × *g* for 10 min. The washed microorganisms were resuspended in 1× PBS and the optical density at 600 nm (OD_600_) of the suspension was measured using a spectrophotometer (Eppendorf, Germany). The concentration of microbial cells was adjusted to 1 × 10^6^ cells/μL of *E. coli* and *S. aureus* and 5 × 10^4^ cells/μL of *C. albicans* for the immune challenge studies. The relevant optimization of microbial concentration has been adjusted based on the previous studies (Chae et al., 2012; Jo et al., 2017; Park et al., 2019).

### Identification and *in silico* Analysis of *TmIKKε*

The *TmIKKε* sequence was retrieved from *T. molitor* RNA sequencing (RNA-seq) (unpublished) and expressed sequence tag (EST) databases. Local-tblastn analysis was performed using *T. castaneum IKKε* amino acid sequence (EEZ99267.2) as a query. The full-length cDNA and deduced amino acid sequences of *Tm*IKKε were determined using the blastx and blastp algorithm, respectively, on the National Center for Biotechnology Information (NCBI) website. Complementary DNA (cDNA) translation and predictions of the deduced protein were analyzed using BioPHP mini tools software (http://www.biophp.org). FGENESH eukaryotic gene prediction was used to predict the *Tm*IKKε open reading frame (ORF) region. The domain architecture of the protein sequences was retrieved using the InterProScan domain analysis program. Representative IKKε protein sequences from other insects were obtained from GenBank and were used for multiple sequence alignments, and percentage identity analysis using Clustal X2.1 (Larkin et al., 2007). A phylogenetic tree was constructed based on the amino acid sequence alignments using the Maximum likelihood method (bootstrap trial set to 1000) with IKKε/TBK1 proteins from representative insects (Supplementary Table 1). The phylogram was analyzed using Tree Explorer view with the Molecular Evolutionary Genetics Analysis (MEGA) version 7.0 program (Kumar et al., 2016) (https://megasoftware.net).

### Sample Collection and Microorganism Challenge

The *TmIKKε* mRNA expression was investigated in different developmental stages of *T. molitor*, eggs (EG), young instar larvae (YL; 10th–12th instar larvae), late-instar larvae (LL; 19th–20th instar larvae), prepupae (PP), 1- to 7-day-old pupae (P1–P7), and 1- to 5-day-old adults (A1–A5). *TmIKKε* mRNA expression was also measured in the different tissues that included integument (IT), hemocytes (HC), GT, fat body (FB), and Malpighian tubules (MT) dissected under a stereoscopic microscope (SMZ645, Nikon, Japan). The tissues were dissected from both LL and adults. Ovary (OV) and testis (TE) were additionally dissected from 5-day-old adults.

To investigate induction patterns of *TmIKKε* mRNA against microbial challenge, 1 × 10^6^ cells/larva of *E. coli* and *S. aureus*, or 5 × 10^4^ cells/larva of *C. albicans* were injected into 12th–15th instar larvae using microinjector with microcapillary. Samples (whole body, HC, GT, and FB) were collected at 3, 6, 9, 12, and 24 h following injection of microorganisms.

### Total RNA Extraction and cDNA Synthesis

The total RNA was isolated from the developmental stages, tissues, and time-course samples using a Clear-S™ Total RNA extraction kit (Invirustech Co., Gwangju, South Korea) according to the manufacturer’s instructions. The total RNA (2 μg) was used as the template to synthesize cDNA using the Oligo(dT)_12–18_ primer on MyGenie96 Thermal Block (Bioneer, South Korea) and AccuPower^®^ RT PreMix (Bioneer) according to the manufacturer’s instructions. The cDNA was stored at −20°C until required.

### Expression and Induction Analysis of the *TmIKKε* mRNA

The relative expression level of *TmIKKε* mRNA was investigated by performing quantitative real-time polymerase chain reaction (qRT-PCR) using an AccuPower^®^ 2× Greenstar™ qPCR Master Mix (Bioneer, Daejeon, Korea) and synthesized cDNAs, and *TmIKKε* gene-specific primers were designed using the Primer 3 plus program (https://primer3plus.com/cgi-bin/dev/primer3plus.cgi) (Supplementary Table 2). The PCR conditions included an initial denaturation at 95°C for 5 min, followed by 40 cycles of denaturation at 95°C for 15 s, and annealing and extension at 60°C for 30 s. The qRT-PCR assays were performed on an AriaMx Real-Time PCR System (Agilent Technologies, United States). The results were analyzed using AriaMx Real-Time PCR software. The 2^–ΔΔCt^ method (Livak and Schmittgen, 2001) was employed to analyze the *TmIKKε* mRNA expression levels. The mRNA expression levels were normalized to those of *T. molitor* ribosomal protein L27a (*TmL27a*), which acted as an internal control. The results represent mean ± standard error (SE) of three biological replicates (3 pools of 20 *T. molitor* larvae).

### *TmIKKε* Gene-Silencing

For the RNA interference (RNAi) experiments of *TmIKKε*, a double-strand RNA (dsRNA) fragment of *TmIKKε* gene was synthesized. Briefly, dsDNA fragment of *TmIKKε* was amplified using PCR with gene-specific primers conjugated with T7 promoter sequences (Supplementary Table 2). The primers were designed using Snapdragon software (https://www.flyrnai.org) to prevent any cross-silencing effects. The primary PCR for the *TmIKKε* gene was carried out using an AccuPower Pfu PCR PreMix (Bioneer) with cDNA and specific primers for the *TmIKKε* gene (Supplementary Table 2). The second PCR was conducted with primers tailed with T7 promoter sequences and 100× dilution of the second PCR products.

Polymerase chain reaction was conducted using an initial denaturation at 94°C for 5 min, followed by 35 cycles of denaturation at 94°C for 30 s, annealing at 53°C for 1 min, and extension at 72°C for 30 s on a MyGenie96 Thermal Block (Bioneer). The PCR products purified using the AccuPrep^®^ PCR Purification Kit (Bioneer) were used to synthesize the dsRNA using the EZ™ T7 High Yield *in vitro* transcription kit (Enzynomics, South Korea). The dsRNA for enhanced green fluorescent protein (ds*EGFP*) synthesized from pEGFP-C1 plasmid DNA as described above acted as a negative control. The dsRNA products were purified using the phenol:chloroform:isoamyl alcohol (PCI) method, precipitated with 5 M ammonium acetate, and washed with 70 and 90% ethanol by centrifugation at 10,000 × *g* for 15 min at 4°C. The dried pellet was resuspended in DNase and RNase-free water. The synthesized dsRNA was stored at −20°C until required. For the knockdown of *TmIKKε* mRNA, 1 μg of dsRNA for *EGFP* and *TmIKKε* were injected into *T. molitor* 10th to 12th instar larvae.

### Mortality Assay

To measure mortality, microorganisms (1 × 10^6^ cells/μl of *E. coli* or *S. aureus*, and 5 × 10^4^ cells/μl of *C. albicans*) were injected into *TmIKKε* gene-silenced *T. molitor* larvae. Dead larvae were counted each day for up to 10 days following the injection of the microorganisms. Ten insect larvae were used for each group in the mortality assay. Each assay was performed in triplicate. Kaplan–Meier method was used to plot cumulative survival curves of larvae after inoculation, the log-rank chi-squared test was used to assess differences in survival between treatments (Goel et al., 2010).

### Effects of *TmIKKε* RNAi on the Expression of Antimicrobial Peptide and NF-κB Genes

To further characterize the function of the *TmIKKε* gene in the humoral innate immune response, the *TmIKKε* silenced individuals were challenged with microorganisms and the expression levels of 14 AMP genes as well as three NF-κB genes were investigated. After treatment of *TmIKKε* dsRNA into *T. molitor* 10th to 12th instar larvae, *E. coli* (1 × 10^6^ cells/larva), *S. aureus* (1 × 10^6^ cells/larva), or *C. albicans* (5 × 10^4^ cells/larva) were injected into *T. molitor* larvae. Twenty-four hours post-injection, over 20 larvae (as a group) were dissected and the samples (HC, GT, and FB) were collected. 1× PBS was used as an injection control. The expression levels of the following 14 AMP genes were measured by qRT-PCR with 14 AMP gene-specific primers (Supplementary Table 2): *TmTenecin 1, 2, 3*, and *4 (TmTene1, 2, 3*, and *4), TmDefensin* and *TmDefensin-like* (*TmDef* and *TmDef-like*), *TmColeoptericin A* and *B* (*TmColeA* and *B*), *TmAttacin 1a, 1b*, and *2* (*TmAtt1a, 1b*, and *2*), *TmCecropin 2* (*TmCec2*), and *TmThaumatin-like protein 1* and *2* (*TmTLP1* and *2*). In addition, the expression profiles of NF-κB genes such as *Tm*Dorsal isoform X1 and X2 (*TmDorX1* and *X2*), and *TmRelish*, were investigated by qRT-PCR. A relative quantitative PCR was performed as mentioned above using the AMP and NF-κB gene specific primers.

### Statistical Analyses

All experiments were carried out in triplicate and the data were subjected to one-way ANOVA. Tukey’s multiple range tests were used to evaluate the difference between groups (*p* < 0.05).

## Results

### Gene Organization, Open Reading Frame and *in silico* Analyses of *TmIKKε*

**FIGURE 1:**
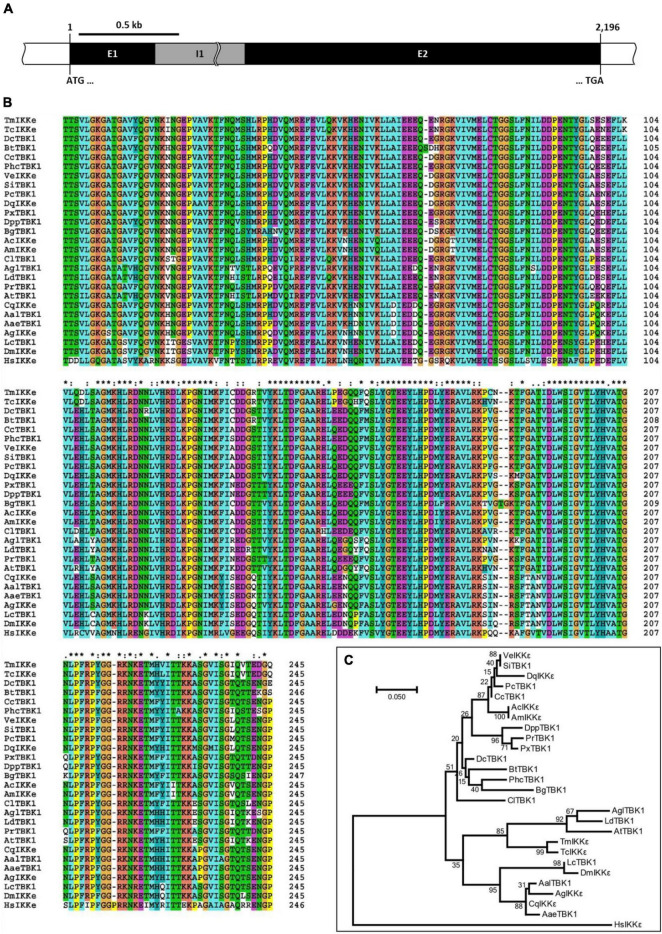
Genomic organization and phylogenetics of the *Tenebrio molitor* IκB kinase ε (*IKKε)* (*TmIKKε)* gene. **(A)** Schematic representation of the *TmIKKε* gene. Two exons (E1 and E2) are separated by a single intron (I1). E1 starts with the translation start codon “ATG,” and E2 ends with the translation stop codon “TGA”. **(B)** Clustal X2-based multiple sequence alignment of IKKε/TBK1 proteins. “*”-Highly conserved residues; “:”-Conserved residues; “.”-Less conserved residues. The residues are colored automatically and represents physico-chemical properties of amino acid residues. **(C)** Maximum likelihood analysis based on the alignment of IKKε/TBK1 amino acid sequences. Bootstrap values (1,000 replicates) are indicated at the nodes.

The organization of the *TmIKKε* gene was deciphered from the *T. molitor* nucleotide database using the *TcIKKε* amino acid sequence as a query in a tBLASTn analysis. *TmIKKε* includes two exons interrupted by a single intron (Figure 1A and Supplementary Figure 1). Exon 1 and exon 2 of *TmIKKε* were 423 bp and 1,773 bp, respectively. The ORF sequence of 2,196 bp starts with the “ATG” initiation codon and ends with the “TGA” stop codon. The ORF sequence of *TmIKKε* encoded a protein of 731 amino acid residues (Supplementary Figure 2). Domain analysis predicted a serine/threonine-protein kinase catalytic domain (residues 12–258), a ubiquitin-like domain (residues 310–387), and a TANK-binding kinase 1 coiled-coil domain 1 (residues 412–653) in *Tm*IKKε protein. A 5′-untranslated region (UTR) sequence of 581 bp and 3′-UTR of 930 bp was also predicted for *TmIKKε*. The 3′-UTR sequence contains a consensus polyadenylation signal (AATAAA) located in 173–178 bp following termination codon (TGA). The *TmIKKε* cDNA sequence and deduced protein sequence have been submitted to GenBank (GenBank ID: MZ708789).

The alignment of the predicted amino acid sequence (restricted to the serine/threonine protein kinase catalytic domain) of *Tm*IKKε with IKKε and serine/threonine protein kinase TBK1 from other known insect IKKs revealed a high degree of conservation (Figure 1B). A phylogenetic tree was constructed using the Clustal X2 alignment file and MEGA 7.0 program to assess the evolutionary position of *Tm*IKKε among the orthologs (Figure 1C). *Homo sapiens* IKKε (*Hs*IKKε) was used as the outgroup in the phylogenetic analysis. Two clear clade divisions were observed. *Tm*IKKε was placed with *Tc*IKKε and TBK1 from other beetles (*Agl*TBK1, *Ld*TBK1, and *At*TBK1). The same clade also placed the IKKε isoforms from the order Diptera in a separate cluster. The high bootstrap values supported the tree topology in this clade. While the mosquito IKKε/TBK1 formed one sub-cluster, the *Drosophila* and blowfly IKKε/TBK1 formed the other sub-cluster. In the second clade, the orthologs of other insect IKKε proteins were placed.

### Expression of *TmIKKε* During Development and in Different Tissues

**FIGURE 2:**
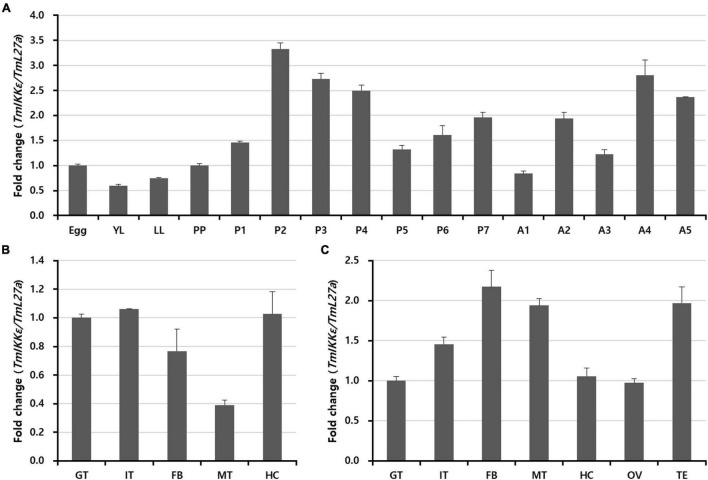
Expression of *TmIKKε* during the developmental stages and in different tissues of *T. molitor*. **(A)** qRT-PCR was used to measure the expression of *TmIKKε* mRNA in the different developmental stages of the insect. YL, early instar larvae; LL, late-instar larvae; PP, pre-pupa; P1–P7, 1- to 7-day-old pupa; A1–A5, 1- to 5-day-old adult. **(B)** Expression of *TmIKKε* mRNA in tissues of *T. molitor* LL as analyzed using real-time polymerase chain reaction (qRT-PCR). **(C)** Expression of *TmIKKε* messenger RNA (mRNA) in the tissues of *T. molitor* adults as analyzed using qRT-PCR. GT, Gut; IT, integument; FB, fat body; MT, Malpighian tubules; HC, hemocytes. *T. molitor* ribosomal protein 27a (*Tm*L27a) was used as an internal control to normalize the concentration of templates between samples. Vertical bars represent mean ± SE (*n* = 20).

In order to examine the *TmIKKε* mRNA expression during development and in different tissues of *T. molitor*, we performed qRT-PCR using SYBR Green dye-binding assay. Developmental expression patterns indicate that *TmIKKε* mRNA was highly expressed in the pupal stage-2, -3, and -4 followed by variable expression in the adult stages (Figure 2A). Further, the expression of *TmIKKε* mRNA was measured in tissues of *T. molitor* late-instar larvae and 5-day old adults using qRT-PCR. In the late-instar larval tissues, the *TmIKKε* mRNA was expressed in a tissue-dependent manner (Figure 2B). *TmIKKε* mRNA was found to be higher (especially in FB, MT, and TE) in the 5-day old adult *T. molitor* (Figure 2C).

### Temporal Expression of *TmIKKε* After Microbial Infection

**FIGURE 3:**
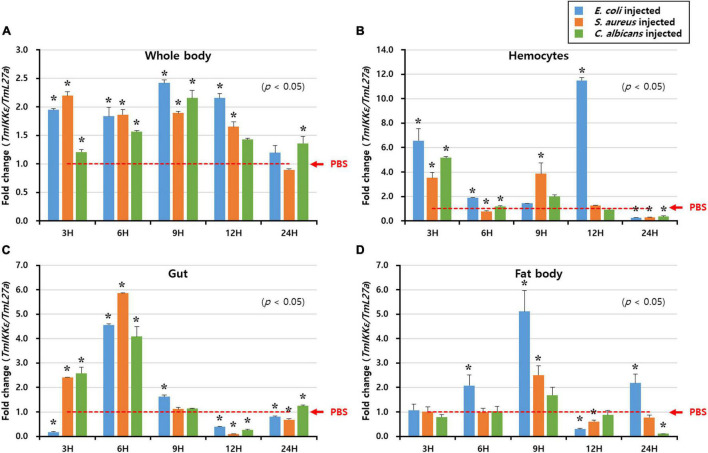
Expression of *TmIKKε* in response to microbial challenge. *Escherichia coli, Staphylococcus aureus*, or *Candida albicans* were injected into 10th to 12th instar larvae, and samples were collected at specific time points (3, 6, 9, 12, and 24 h). *TmIKKε* mRNA expression in the whole body **(A)**, hemocytes **(B)**, gut **(C)**, and fat body **(D)** of *T. molitor* larvae. The *TmIKKε* mRNA expression was normalized to that in the phosphate-buffered saline (PBS) group. *Tm*L27a was used as an internal control. Data represent the mean ± SE. Significance is set at *p* < 0.05. *Denotes significant difference over the control.

To understand the biological function of *TmIKKε* in innate immunity of *T. molitor*, the mRNA expression levels were monitored at different time-points (3, 6, 9, 12, and 24 h) in whole-larvae, HC, GT, and FB after exposure to PBS (as injection controls), and microorganisms (*E. coli*, *S. aureus*, and *C. albicans*) challenge (Figure 3). The expression level of *TmIKKε* at various time points was analyzed relative to PBS control. In the whole body, *TmIKKε* mRNA was induced early at 3 h and declined at 6, 9, and 12 h post-injection of microorganisms towards the level of PBS-injected control. At 24 h, the expression of *TmIKKε* mRNA was found to be lower compared to the expression at 3 h (Figure 3A). In hemocytes, *TmIKKε* mRNA was induced at 3 and 12 h post-injection by *E. coli*. *S. aureus* induced *TmIKKε* mRNA early at 3 and 9 h post-challenge (Figure 3B). But in the gut tissue, expression of *TmIKKε* mRNA was drastically high at 6 h-post-injection of *S. aureus*, *E. coli*, and *C. albicans* (Figure 3C). Expression of *TmIKKε* mRNA in the FB was higher at 9 h post-injections with *E. coli* and is decreased at 12 hpi relative even to 3 hpi (Figure 3D).

### Effects of *TmIKKε* RNAi on Larval Survivability

**FIGURE 4:**
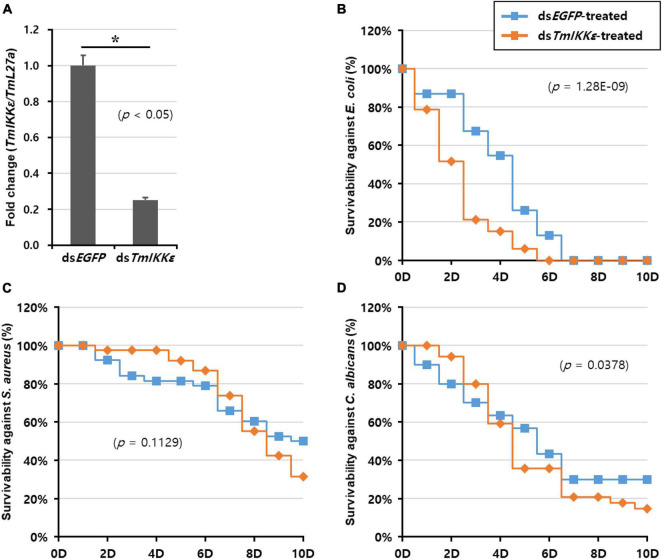
*TmIKKε* gene-silencing and larval survival against pathogenic challenge. **(A)** RNA interference (RNAi) efficiency of *TmIKKε* mRNA after ds*TmIKKε* injection into *T. molitor* measured using qRT-PCR. The *EGFP* dsRNA-treated larvae served as a negative control. Larval survival was measured until 10 days after injection with *E. coli* **(B)**, *S. aureus* **(C)**, and *C. albicans* **(D)**. The Kaplan–Meier survival plots (log-rank chi-squared test) were used to analyze the larval mortality. *Denotes significant difference over control.

To further substantiate the function of *TmIKKε* in the host immunity against pathogens, we silenced *TmIKKε* mRNA by synthesizing dsRNA and injected it into the *T. molitor* larvae. The silencing of *TmIKKε* mRNA was compared relative to the injection of ds*EGFP* (as negative control) to a separate set of larvae. The RNAi efficiency of *TmIKKε* was found to be approximately 75% relative to the ds*EGFP* control in the whole body of the larvae (Figure 4A). In addition, the tissue-specific knockdown efficiency by injecting *TmIKKε* dsRNA was investigated, which showed the results that the expression of *TmIKKε* was significantly down-regulated by ds*TmIKKε*-treatment in all tissues including FB (86%), GT (68%), HC (89%), and integuments (84%) (Supplementary Figure 3).

The survival of the larvae was recorded for 10 days after injection of *E. coli*, *S. aureus*, and *C. albicans* to *TmIKKε-*silenced larvae. After *E. coli* challenge to *TmIKKε-*silenced larvae, the mortality observed was 100% at 6-day post-challenge but was not significant compared to *dsEGFP-*treated larvae (log-rank chi-squared test; *p* = 1.28E-09) (Figure 4B). The mortality of *TmIKKε-*silenced larvae was 70% at 10-day post-challenge with *S. aureus* (log-rank chi-squared test; *p* = 0.1129) (Figure 4C). After infection with the fungus, *C. albicans*, the mortality was close to 90% in *TmIKKε-*silenced larvae (log-rank chi-squared test; *p* = 0.0378) (Figure 4D). The results indicate that depletion of *TmIKKε* caused increased larval mortality against *E. coli*, not *S. aureus* and *C. albicans.*

### Antimicrobial Peptide Expression Levels in *TmIKKε* Knockdown *T. molitor* Larvae

**FIGURE 5:**
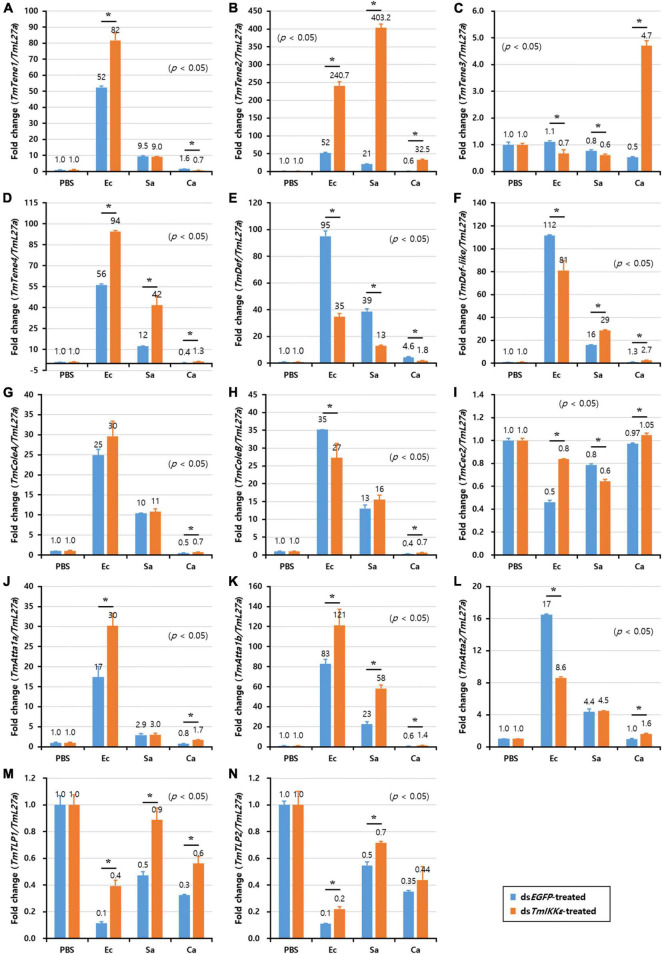
The antimicrobial peptides (AMPs) expression levels in *TmIKKε-*knockdown *T. molitor* larval hemocytes upon microorganism challenge. *E. coli* (Ec), *S. aureus* (Sa), or *C. albicans* (Ca) were injected into *TmIKKε*-silenced *T. molitor* larvae. The transcriptional expression levels of *TmTene1* **(A)**, *TmTene2* **(B)**, *TmTene3* **(C)**, *TmTene4* **(D)**, *TmDef* **(E)**, *TmDef-like* **(F)**, *TmColeA* **(G)**, *TmColeB* **(H)**, *TmCec2* **(I)**, *TmAtta1a* **(J)**, *TmAtta1b* **(K)**, *TmAtta2* **(L)**, *TmTLP1* **(M)**, and *TmTLP2* **(N)** were measured using qRT-PCR. *EGFP* dsRNA was used as a silencing control, and *TmL27a* was used as an internal control. Data represent the mean ± SE of three independent biological replicates. Asterisks indicate significant differences between dsTmSpz5- and ds*EGFP*-treated groups when compared using Student’s *t*-test (*p* < 0.05).

In order to investigate the requirement of *TmIKKε* gene in the regulation of AMP production in immune organs (hemocytes, gut, and fat body) of *T. molitor* larvae, we injected gram-negative bacteria *E. coli*, gram-positive bacteria *S. aureus* or fungus *C. albicans* into *TmIKKε* knockdown *T. molitor* larvae. The transcriptional expression levels of fourteen *T. molitor* AMP genes were measured in *TmIKKε* knockdown individuals in comparison with ds*EGFP*-treated group. In the hemocytes of *T. molitor* larvae, the expression of *TmTene2* was significantly upregulated in *TmIKKε* knockdown individuals post-inoculation with *E. coli*, *S. aureus*, and *C. albicans* (Figure 5B). *TmTene1* (Figure 5A) and *TmTene3* (Figure 5C) expression were also upregulated post-inoculation of *E. coli* and *C. albicans*, respectively. Further, the expression of *TmAtta1a* was upregulated post-inoculation of *E. coli* (Figure 5J) and *TmAtta1b* upregulated post-inoculation of *E. coli* and *S. aureus* (Figure 5K) in *TmIKKε* knockdown individuals. The upregulation of AMPs in response to *TmIKKε* gene knockdown suggests negative regulation during the pathogenic challenge.

**FIGURE 6:**
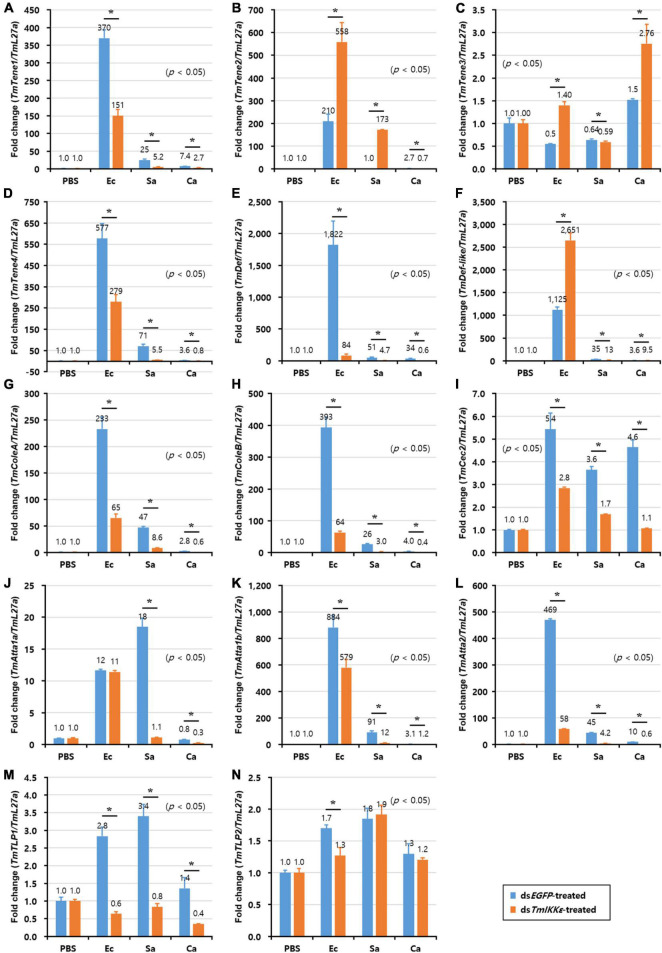
AMP expression levels in *TmIKKε-*knockdown *T. molitor* larval gut upon microorganism challenge. *E. coli* (Ec), *S. aureus* (Sa), or *C. albicans* (Ca) were injected into *TmIKKε*-silenced *T. molitor* larvae. The transcriptional expression levels of *TmTene1* **(A)**, *TmTene2* **(B)**, *TmTene3* **(C)**, *TmTene4* **(D)**, *TmDef* **(E)**, *TmDef-like* **(F)**, *TmColeA* **(G)**, *TmColeB* **(H)**, *TmCec2* **(I)**, *TmAtta1a* **(J)**, *TmAtta1b* **(K)**, *TmAtta2* **(L)**, *TmTLP1* **(M)**, and *TmTLP2* **(N)** were measured using qRT-PCR. *EGFP* dsRNA was used as a silencing control, and *TmL27a* was used as an internal control. Data represent the mean ± SE of three independent biological replicates. Asterisks indicate significant differences between dsTmSpz5- and ds*EGFP*-treated groups when compared using Student’s *t*-test (*p* < 0.05).

Alternatively, in the gut, the expression of eight AMP genes including *TmTene1* (Figure 6A), *TmTene4* (Figure 6D), *TmDef* (Figure 6E), *TmColeA* (Figure 6G), *TmColeB* (Figure 6H), *TmAtta1a* (Figure 6J), *TmAtta1b* (Figure 6K), and *TmAtta2* (Figure 6L) out of the fourteen AMP genes were significantly decreased in *TmIKKε* knockdown individuals. On the other hand, the expression of only two AMP genes including *TmTene2* (Figure 6B) and *TmDef-like* (Figure 6F) was significantly upregulated by *TmIKKε* RNAi. Overall, in the gut of *T. molitor* larvae, *TmIKKε* RNAi leads to decreased transcriptional regulation of most AMPs and might be putatively involved in the survival of the larvae against pathogenic stress. We also noticed the downregulation of *TmIKKε* transcripts in the gut tissue following systemic injection of ds*TmIKKε*. Moreover, in the silenced individuals, eight AMPs were downregulated suggesting putative role in gut immunity.

**FIGURE 7:**
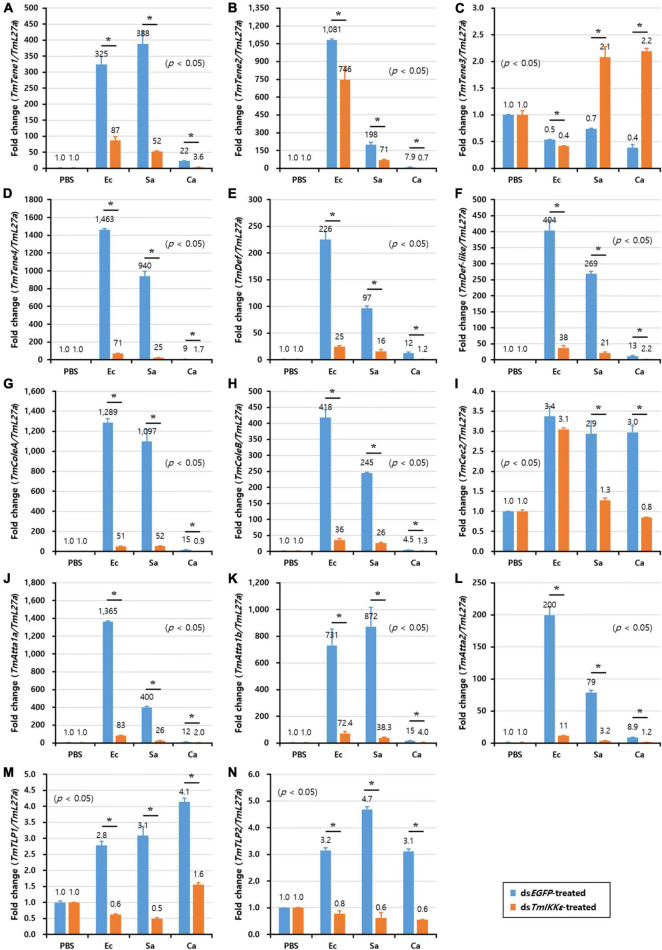
AMP expression levels in *TmIKKε-*knockdown *T. molitor* larval fat body upon microorganism challenge. *E. coli* (Ec), *S. aureus* (Sa), or *C. albicans* (Ca) were injected into *TmIKKε*-silenced *T. molitor* larvae. The transcriptional expression levels of *TmTene1* **(A)**, *TmTene2* **(B)**, *TmTene3* **(C)**, *TmTene4* **(D)**, *TmDef* **(E)**, *TmDef-like* **(F)**, *TmColeA* **(G)**, *TmColeB* **(H)**, *TmCec2* **(I)**, *TmAtta1a* **(J)**, *TmAtta1b* **(K)**, *TmAtta2* **(L)**, *TmTLP1* **(M)**, and *TmTLP2* **(N)** were measured using qRT-PCR. *EGFP* dsRNA was used as a silencing control, and *TmL27a* was used as an internal control. Data represent the mean ± SE of three independent biological replicates. Asterisks indicate significant differences between dsTmSpz5- and ds*EGFP*-treated groups when compared using Student’s *t*-test (*p* < 0.05).

In *T. molitor* FB, the expression of twelve AMP genes including *TmTene1* (Figure 7A), *-2* (Figure 7B) and *-4* (Figure 7D), *TmDef* (Figure 7E) and *-2* (Figure 7F), *TmColeA* (Figure 7G) and *-2* (Figure 7H), *TmAtta1a* (Figure 7J), *-1b* (Figure 7K), and *-2* (Figure 7L), *TmTLP1* (Figure 7M), and *-2* (Figure 7N), out of fourteen AMP genes were significantly decreased by *TmIKKε* RNAi. Downregulation of AMPs in *TmIKKε* knockdown individuals after challenge with microorganisms ascertains the role of *TmIKKε* in the innate immunity of the insect.

### Effects of *TmIKKε* RNAi on the Expression of *Tenebrio* NF-κB Genes

**FIGURE 8:**
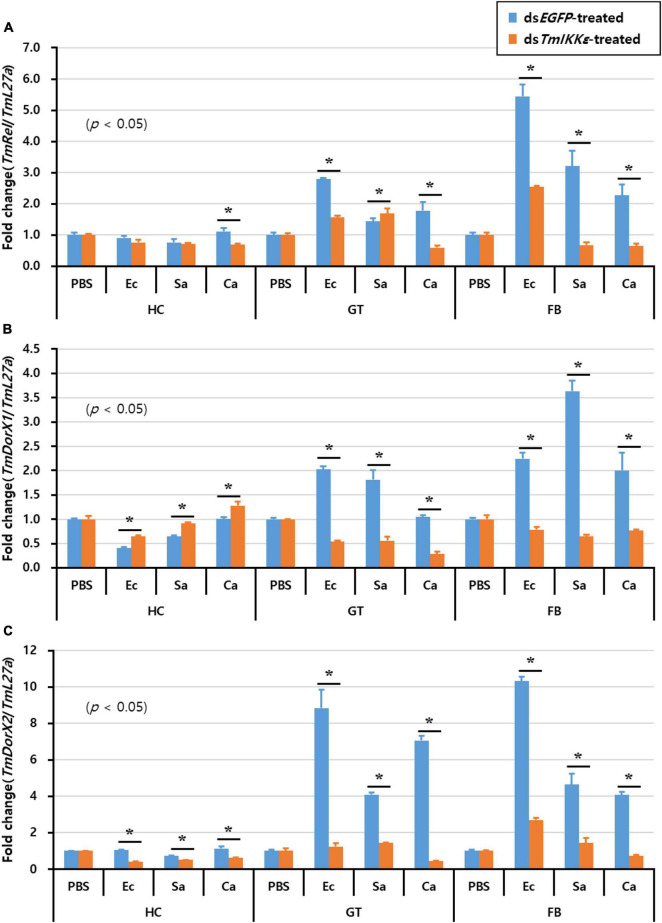
Tissue-specific induction patterns of *Tenebrio* NF-κB genes in *TmIKKε* dsRNA-treated *T. molitor* larvae against various pathogens. **(A)** Induction of *TmRelish* mRNA in the hemocytes, gut, and fat body of *T. molitor* larvae. **(B)** Induction of *TmDorX1* mRNA in the hemocytes, gut, and fat body of *TmIKKε*-silenced *T. molitor* larvae. **(C)** Induction of *TmDorX2* mRNA in the hemocytes, gut, and fat body of *TmIKKε*-silenced *T. molitor* larvae. Asterisks indicate significant differences between dsTmSpz5- and ds*EGFP*-treated groups when compared using Student’s *t*-test (*p* < 0.05).

Furthermore, to understand the effect of *TmIKKε* RNAi on expression of *Tenebrio* NF-κB genes, *TmIKKε*-silenced *T. molitor* larvae were challenged with microorganisms and the expression patterns of *Tenebrio* NF-κB genes such as *TmRelish*, *TmDorX1*, and *TmDorX2* were investigated at 24 h by qPCR analysis. The results showed that the mRNA level of three NF-κB genes was dramatically decreased by *TmIKKε* RNAi in the FB of *T. molitor* larva. In addition, the expression of *TmDorX1* and *TmDorX2* transcripts were significantly decreased by *TmIKKε* RNAi in the GT (Figure 8).

## Discussion

The Toll and IMD pathways constitute an important defense arsenal to protect insects from non-self-discriminating pathogens. Our research group has been successful in elucidating key genes of the Toll and IMD intracellular pathways, which are relevant in the context of humoral immunity in the coleopteran pest *T. molitor* (Patnaik et al., 2013, 2014; Tindwa et al., 2013). Our focus has been on the transcriptional activation of AMPs elicited by diverse groups of microorganisms and mediated through the Toll/IMD signaling cascade (Jo et al., 2017, 2019; Keshavarz et al., 2019, 2020a,b, 2020d; Park et al., 2019; Ali Mohammadie Kojour et al., 2020; Edosa et al., 2020a,b; Ko et al., 2020). The IKK family of proteins act upstream of the NF-κB factor Relish in the IMD pathway by phosphorylating Relish and regulating the transcriptional activation of AMP genes. The involvement of the IKK isoforms *IKKβ* (*ird5* in *Drosophila*) and *IKKγ* (*Kenny* in *Drosophila*) in phosphorylation of Relish has been described earlier (Erturk-Hasdemir et al., 2009; Kleino and Silverman, 2014). The IKKε isoform of the IKK family of proteins encodes a serine-threonine kinase that has been implicated in NF-κB activation. The isoform also forms an essential component of the interferon regulatory factor 3 (IRF3) signaling pathway (Fitzgerald et al., 2003; Seccareccia et al., 2014; Dubois et al., 2018). IKKε in the black carp (*Mylopharyngodon piceus*) was functionally characterized to participate in activating the expression of interferons in zebrafish and epithelioma papulosum cyprini (EPC) cells (Qu et al., 2015). TBK1, which is structurally identical to other IKK proteins, may also have a putative role as an important immunoregulator for IRF3 and IFNγ induction in chickens (Wang et al., 2017). However, the functional characterization of the IKKε homolog in insects is less described. In this study, we characterized the *IKKε* isoform in the coleopteran pest *T. molitor* by identifying its sequence, tissue distribution, and possible role in humoral immunity by studying the transcriptional regulation of 14 *T. molitor* AMP genes after microorganism challenge. We have also examined the expression of downstream NF-κB factors, including *TmDorX1* and *TmDorX2*, which are involved in the Toll signaling pathway, and Relish, which is involved in the IMD signaling pathway, under *TmIKKε*-silenced conditions. Our results have improved understanding of the putative regulatory pathway involving *Tm*IKKε.

The induction pattern of *TmIKKε* against injection of representative gram-negative bacteria, gram-positive bacteria, and fungi indicated that *TmIKKε* mRNA expression was mainly induced by *E. coli* challenge in the hemocytes. Furthermore, the knockdown of *TmIKKε* transcripts resulted in larval death upon *E. coli* challenge. The findings suggest that *TmIKKε* may be putatively involved in the defense against gram-negative bacteria. Interestingly, the *TmIKKε* transcript was significantly induced by *S. aureus* in hemocytes. This result may indicate the involvement of *Tm*IKKε in the canonical IMD pathway. Further, the role of *S. aureus* in the activation of the Toll signaling cascade is well established in insects, including *T. molitor* (Patnaik et al., 2014). However, in the gut defense system of *D. melanogaster*. IMD pathway is required for clearance of *S. aureus*, possibly independently from AMP expression and *via* Duox system that produces reactive oxygen species (ROS) (Hori et al., 2018).

The transcriptional regulation of fourteen AMP genes in *T. molitor* in *TmIKKε-*silenced condition was investigated in hemocytes, gut, and fat body tissues post-injection with gram-negative bacteria *E. coli*, gram-positive bacteria *S. aureus*, and the fungus *C. albicans*. Interestingly, most AMP genes were not positively affected by *TmIKKε* RNAi in hemocytes, excepting to some extent *TmDef*. Other critical AMP genes were mostly negatively regulated after microorganisms challenge in *TmIKKε-*silenced individuals. Further, the inconsequential role of *TmIKKε* knockdown on the activation of the NF-κB genes including *TmRelish*, *TmDorX1*, and *TmDorX2* suggest that *Tm*IKKε is not required for AMP production in hemocytes. Contrastingly, in the gut, seven AMP genes were significantly downregulated by *TmIKKε* dsRNA-treatment post-injection of *E. coli*. We also find that the *TmDorX1* and *TmDorX2* mRNA (NF-κB regulator of Toll signaling pathway) were critically downregulated in the gut in *TmIKKε-*silenced individuals. This possibly suggests that *Tm*IKKε regulates the transcriptional activation of seven AMP genes in the host gut in response to the systemic infection of microorganisms. Studies in mosquitoes highlight the promiscuous intervention of hemocytes in the activation of the anti-plasmodial gut immune system (Ramirez et al., 2014; Castillo et al., 2017). These pieces of evidence suggest the relationship between systemic infections and gut innate immune system.

In the fat body tissue of *T. molitor* larvae, the expression of ten AMP genes was downregulated and all the three NF-κB genes were significantly affected by *TmIKKε* RNAi in response to microbial challenge. These results indicate that *Tm*IKKε is a key regulator for Toll and IMD pathways in the fat body of *T. molitor* larvae. Cross-talk between Toll and IMD pathways is proposed in *D. melanogaster* (Tanji et al., 2007), *T. castaneum* (Yokoi et al., 2012a,b), *P. stali* (Nishide et al., 2019), and *T. molitor* (Ko et al., 2020). It was also suggested that several AMP genes were co-regulated by those pathways in *D. melanogaster* (De Gregorio et al., 2002). Further, DmIKKε phosphorylates DIAP1 leading to DIAP1 degradation and apoptosis in development downstream of IMD. In *Drosophila*, the function of DmIKKε is not especially Toll or IMD related, particularly for its role in IMD activation (Kuranaga et al., 2006; Oshima et al., 2006). In addition, the activation of Toll and IMD pathways is totally dependent on invading microbes. For instance, the gram-positive bacteria including *Micrococcus luteus*, *Bacillus subtilis*, *Bacillus megaterium, Enterobacter cloacae*, and fungi such as *Beauveria bassiana*, *Saccharomyces cerevisiae*, *Metarhizium anisopliae*, and *Geotrichum candidum* activate the IMD pathway (Hedengren-Olcott et al., 2004). *Drosophila* Cecropin A was induced by the gram-positive bacteria *M. luteus* and *S. aureus* independent of the NF-κB factor Relish. The transcriptional activation of AMPs such as *Cecropin A1* and *Cecropin A2* in response to *M. luteus* infection required Relish and Dif, respectively. Even, it is well known that the Tenecin 3 protects *T. molitor* against infection by the fungus *Beauveria bassiana* exemplified by increased larval survivability (Maistrou et al., 2018). Contrastingly, our results showed that the *TmIKKε* RNAi does not mainly affect the expression of Tenecin 3 gene and larval mortality against fungal infection.

Taken together, *Tm*IKKε plays a critical function in the production of nine AMPs in the fat body by regulating both Toll (*Tm*DorX1 and -X2) and IMD (*Tm*Relish) pathways. Interestingly, seven AMP genes were positively regulated by *Tm*IKKε RNAi in the gut, carefully suggesting that the systemic infection may positively regulate AMP production in the gut through the Toll (*Tm*DorX1 and -X2) pathway.

## Data Availability Statement

The original contributions presented in the study are included in the article/Supplementary Material, further inquiries can be directed to the corresponding authors.

## Author Contributions

YH and YJ: conceptualization, methodology, supervision, and project administration. YH: software, validation, resources, and funding acquisition. HK and YJ: formal analysis and visualization. HK, KP, CK, and HJ: investigation. HK, KP, and CK: data curation. HK, YH, YJ, and BP: writing – original draft preparation. BP, SB, and YL: writing – review and editing. All authors have read and agreed to the published version of the manuscript.

## Conflict of Interest

The authors declare that the research was conducted in the absence of any commercial or financial relationships that could be construed as a potential conflict of interest.

## Publisher’s Note

All claims expressed in this article are solely those of the authors and do not necessarily represent those of their affiliated organizations, or those of the publisher, the editors and the reviewers. Any product that may be evaluated in this article, or claim that may be made by its manufacturer, is not guaranteed or endorsed by the publisher.
