# Supplementary material for: TmIKKε Is Required to Confer Protection Against Gram-Negative Bacteria, E. coli by the Regulation of Antimicrobial Peptide Production in the Tenebrio molitor Fat Body
Source: Front Physiol. 2022 Jan 7;12:758862. doi: 10.3389/fphys.2021.758862 (PMC8777057; doi:10.3389/fphys.2021.758862)
Supplement: Supplementary file 1 [file Data_Sheet_1.DOCX]

***Tm*IKKε is required to confer protection against Gram-negative bacteria, *E. coli* by the regulation of AMP production in the *Tenebrio molitor* fat body**

Hye Jin Ko ^1^, Bharat Bhusan Patnaik ^2^, Ki Beom Park ^1^, Chang Eun Kim ^1^, Snigdha Baliarsingh ^2^, Ho Am Jang ^1^, Yong Seok Lee ^3^, Yeon Soo Han ^1,*^, and Yong Hun Jo ^1,*^

^1^ Department of Applied Biology, Institute of Environmentally-Friendly Agriculture (IEFA), College of Agriculture and Life Sciences, Chonnam National University, Gwangju 61186, Korea

^2^ P.G. Department of Bio-Sciences and Bio-Technology, Fakir Mohan University, Nuapadhi, Balasore, Odisha 756089, India

^3^ Department of Biology, College of Natural Sciences, Soonchunhyang University, 22 Soonchunhyangro, Shinchang-Myeon, Asan, Chungchungnam-do 31538, Korea

^*^ Correspondence:

Yon Hun Jo (yhun1228@jnu.ac.kr) and Yeon Soo Han (hanys@jnu.ac.kr)

**Supplementary Data**

Supplementary Table 1. Gene information of phylogenetic analysis in the study

| **Abb.** | **Species** | **Name** | **Accession No.** |
| --- | --- | --- | --- |
| TmIKKε | *T. molitor* | inhibitor of nuclear factor-kappa-B kinase subunit epsilon | MZ708789 |
| TcIKKε | *T. castaneum* | inhibitor of nuclear factor-kappa-B kinase subunit epsilon | EFA07517.1 |
| *Agl*TBK1 | *A. glabripennis* | serine/threonine protein kinase TBK1 | XP_018575342.1 |
| *Ld*TBK1 | *L. decemlineata* | serine/threonine protein kinase TBK1-like | XP_023014530.1 |
| *At*TBK1 | *A. tumida* | serine/threonine protein kinase TBK1 | XP_019866674.1 |
| *Phc*TBK1 | *P. humanus corporis* | serine/threonine protein kinase TBK1 putative | XP_002428501.1 |
| *Pc*TBK1 | *P. canadensis* | serine/threonine protein kinase TBK1 isoform X1 | XP_014609133.1 |
| *Cc*TBK1 | *C. cinctus* | serine/threonine protein kinase TBK1 isoform X1 | XP_015593798.1 |
| *Dq*IKKε | *D. quadriceps* | inhibitor of nuclear factor-kappa-B kinase subunit epsilon | XP_014483559.1 |
| *Ve*IKKε | *V. emeryi* | inhibitor of nuclear factor-kappa-B kinase subunit epsilon | XP_011867588.1 |
| *Si*TBK1 | *S. invicta* | serine/threonine protein kinase TBK1 | XP_011170700.1 |
| *Dc*TBK1 | *D. citri* | serine/threonine protein kinase TBK1 isoform X1 | XP_008475603.1 |
| *Bt*TBK1 | *B. tabaci* | serine/threonine protein kinase TBK1 | XP_018901849.1 |
| *Ac*IKKε | *A. cerana* | inhibitor of nuclear factor-kappa-B kinase subunit epsilon | XP_016914720.1 |
| *Am*IKKε | *A. mellifera* | inhibitor of nuclear factor-kappa-B kinase subunit epsilon | XP_396937.4 |
| *Bg*TBK1 | *B. germanica* | serine/threonine protein kinase TBK1 | PSN34189.1 |
| *Cl*TBK1 | *C. lectularius* | serine/threonine protein kinase TBK1-like | XP_014248726.1 |
| *Aal*TBK1 | *A. albopictus* | serine/threonine protein kinase TBK1 isoform X2 | XP_019550623.1 |
| *Aae*TBK1 | *A. aegypti* | serine/threonine protein kinase TBK1 | XP_021702308.1 |
| *Dpp*TBK1 | *D. plexippus* | serine/threonine protein kinase TBK1 | OWR41799.1 |
| *Pr*TBK1 | *P. rapae* | serine/threonine protein kinase TBK1 | XP_022117350.1 |
| *Px*TBK1 | *P. xuthus* | serine/threonine protein kinase TBK1 | XP_013163148.1 |
| *Cq*IKKε | *C. quinquefasciatus* | inhibitor of kappa b kinase epsilon | XP_001848400.1 |
| *Ag*IKKε | *A. gambiae* | AGAP009565-PA partial | XP_318573.3 |
| *Lc*TBK1 | *L. cuprina* | serine/threonine protein kinase TBK1 | XP_023299337.1 |
| *Dm*IKKε | *D. melanogaster* | I-kappaB kinase epsilon isoform B | NP_724278.1 |
| *Hs*IKKε | *H. sapiens* | inhibitor of nuclear factor-kappa-B kinase subunit epsilon isoform 2 | NP_001180251.1 |

Supplementary Table 2. Primers used in the study

| **Name** | **Primer sequences** |
| --- | --- |
| *Tm*IKKε_cloning_Fw  *Tm*IKKε_cloning_Rv | 5′-GAAGCACCTTCGGGACAATA-3′  5′-CTTCTCCAAGAGGTGGATGC-3′ |
| *Tm*IKKε_qPCR_Fw  *Tm*IKKε_qPCR_Rv | 5′-ACAACGTTTTGGTGGTTCC-3′  5′-AGCGTTTTTGGCTTGAGCTG-3′ |
| *Tm*IKKε_T7_Fw  *Tm*IKKε_T7_Rv | 5′-TAATACGACTCACTATAGGGT GAAGCACCTTCGGGACAATA-3′  5′-TAATACGACTCACTATAGGGT CTTCGTGTCAGCTTCCAACA-3′ |
| EGFP-T7-Fw  EGFP-T7-Rv | 5′-TAATACGACTCACTATAGGGT CGTAAACGGCCACAAGTTC-3′  5′-TAATACGACTCACTATAGGGT TGCTCAGGTAGTGTTGTCG-3′ |
| *Tm*Tenecin-1_qPCR_Fw  *Tm*Tenecin-1_qPCR_Rv | 5′-CAGCTGAAGAAATCGAACAAGG-3′  5′-CAGACCCTCTTTCCGTTACAGT-3′ |
| *Tm*Tenecin-2_qPCR_Fw  *Tm*Tenecin-2_qPCR_Rv | 5′-CAGCAAAACGGAGGATGGTC-3′  5′-CGTTGAAATCGTGATCTTGTCC-3′ |
| *Tm*Tenecin-3_qPCR_Fw  *Tm*Tenecin-3_qPCR_Rv | 5′-GATTTGCTTGATTCTGGTGGTC-3′  5′-CTGATGGCCTCCTAAATGTCC-3′ |
| *Tm*Tenecin-4_qPCR_Fw  *Tm*Tenecin-4_qPCR_Rv | 5′-GGACATTGAAGATCCAGGAAAG-3′  5′-CGGTGTTCCTTATGTAGAGCTG-3′ |
| *Tm*Defensin_qPCR_Fw  *Tm*Defensin_qPCR_Rv | 5′-AAATCGAACAAGGCCAACAC-3′  5′-GCAAATGCAGACCCTCTTTC-3′ |
| *Tm*Defensin-like_qPCR_Fw  *Tm*Defensin-like_qPCR_Rv | 5′-GGGATGCCTCATGAAGATGTAG-3′  5′-CCAATGCAAACACATTCGTC-3′ |
| *Tm*Coleoptericin-A_qPCR_Fw  *Tm*Coleoptericin-A_qPCR_Rv | 5′-GGACAGAATGGTGGATGGTC-3′  5′-CTCCAACATTCCAGGTAGGC-3′ |
| *Tm*Coleoptericin-B_qPCR_Fw  *Tm*Coleoptericin-B_qPCR_Rv | 5′-CAGCTGTTGCCCACAAAGTG-3′  5′-CTCAACGTTGGTCCTGGTGT-3′ |
| *Tm*Attacin-1a_qPCR_Fw  *Tm*Attacin-1a_qPCR_Rv | 5′-AAAGTGGTCCCCACCGATTC-3′  5′-GCGCTGAATGTTTTCGGCTT-3′ |
| *Tm*Attacin-1b_qPCR_Fw  *Tm*Attacin-1b_qPCR_Rv | 5′-GAGCTGTGAATGCAGGACAA-3′  5′-CCCTCTGATGAAACCTCCAA-3′ |
| *Tm*Attacin-2_qPCR_Fw  *Tm*Attacin-2_qPCR_Rv | 5′-AACTGGGATATTCGCACGTC-3′  5′-CCCTCCGAAATGTCTGTTGT-3′ |
| *Tm*Cecropin-2_qPCR_Fw  *Tm*Cecropin-2_qPCR_Rv | 5′-TACTAGCAGCGCCAAAACCT-3′  5′-CTGGAACATTAGGCGGAGAA-3′ |
| *TmTLP-1*_qPCR_Fw  *TmTLP-1*_qPCR_Rv | 5′-CTCAAAGGACACGCAGGACT-3′  5′-ACTTTGAGCTTCTCGGGACA-3′ |
| *TmTLP-2*_qPCR_Fw  *TmTLP-2*_qPCR_Rv | 5′-CCGTCTGGCTAGGAGTTCTG-3′  5′-ACTCCTCCAGCTCCGTTACA-3′ |
| *Tm*DorX1_qPCR_Fw  *Tm*DorX1_qPCR_Rv | 5′-AGCGTTGAGGTTTCGGTATG-3′  5′-TCTTTGGTGACGCAAGACAC-3′ |
| *Tm*DorX2_qPCR_Fw  *Tm*DorX2_qPCR_Rv | 5′-ACACCCCCGAAATCACAAAC-3′  5′-TTTCAGAGCGCCAGGTTTTG-3′ |
| *Tm*Relish_qPCR_Fw  *Tm*Relish_qPCR_Rv | 5′-AGCGTCAAGTTGGAGCAGAT-3′  5′-GTCCGGACCTCAAGTGT-3′ |
| *Tm*L27a_qPCR_Fw  *Tm*L27a_qPCR_Rv | 5′-TCATCCTGAAGGCAAAGCTCCAGT-3′  5′-AGGTTGGTTAGGCAGGCACCTTTA-3′ |

※ Underline indicates T7 promotor sequences

**Figures**


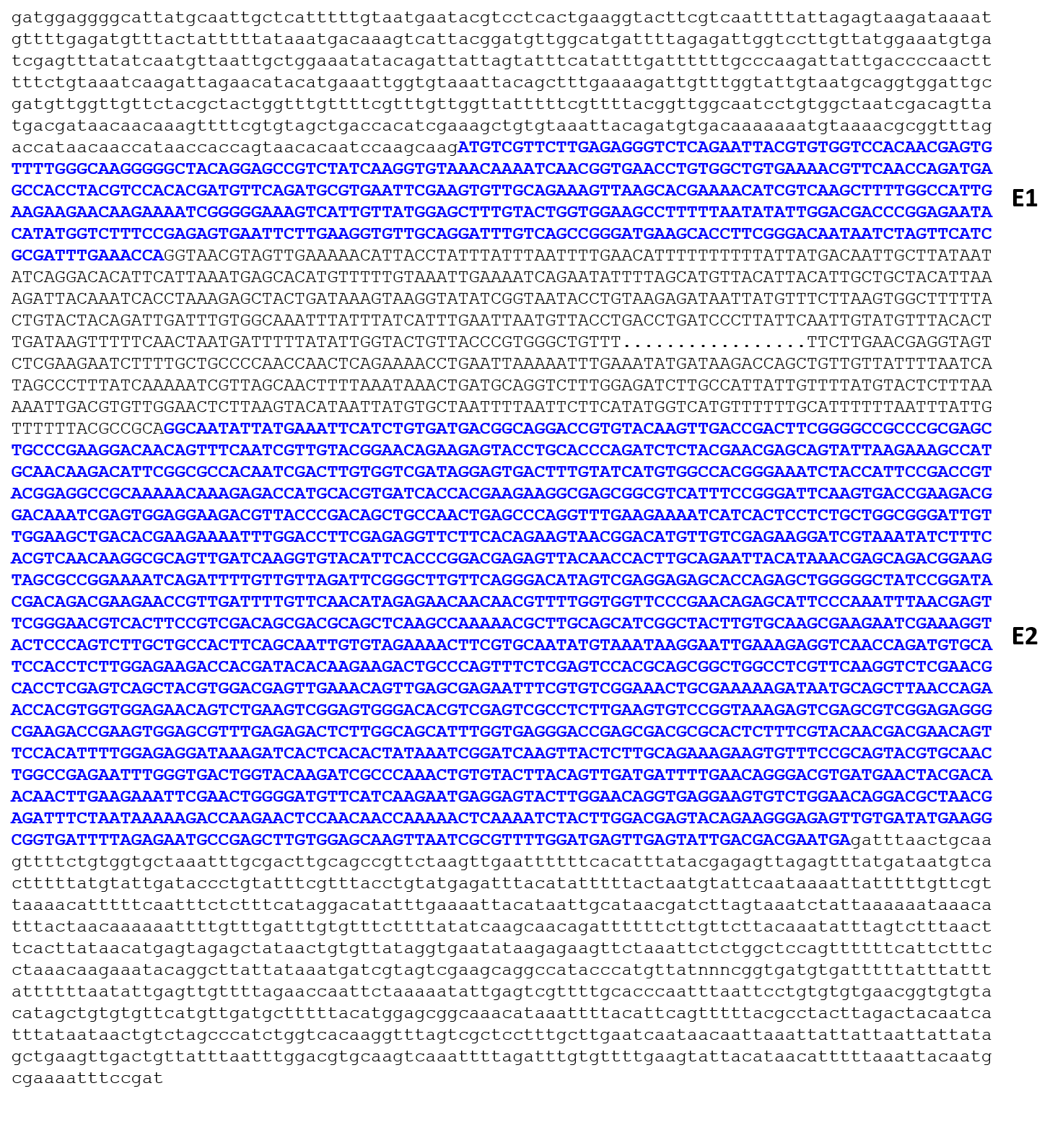


**Supplementary Figure 1**. Genomic organization of the *T. molitor* *IKKε* (*TmIKKε)* gene. The sequence of *TmIKKε* gene with the open reading frame region is shown capital letters. *TmIKKε* comprises a nucleotide sequence of 2,196 bp starting from the initiation codon (ATG) until the termination codon (TGA) within two exons.


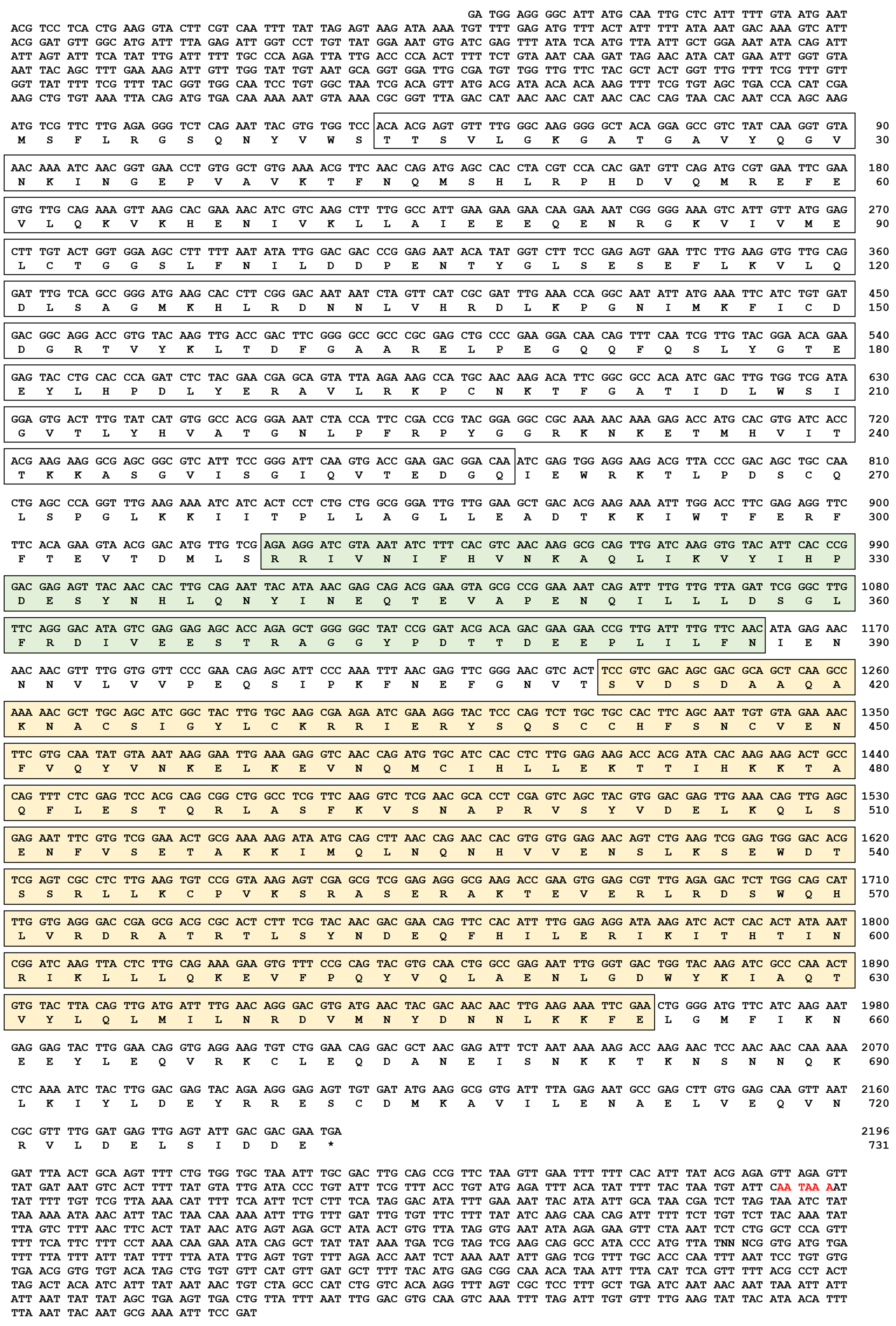


**Supplementary Figure 2**. Nucleotide and deduced amino acid sequences of *TmIKKε.* The nucleotides and amino acids are numbered from the translation start codon. The serine/threonine protein kinase catalytic domain is shown in an open box. Green and yellow boxes indicate ubiquitin-like domain and TANK-binding kinase 1 coiled-coil domain 1, respectively.


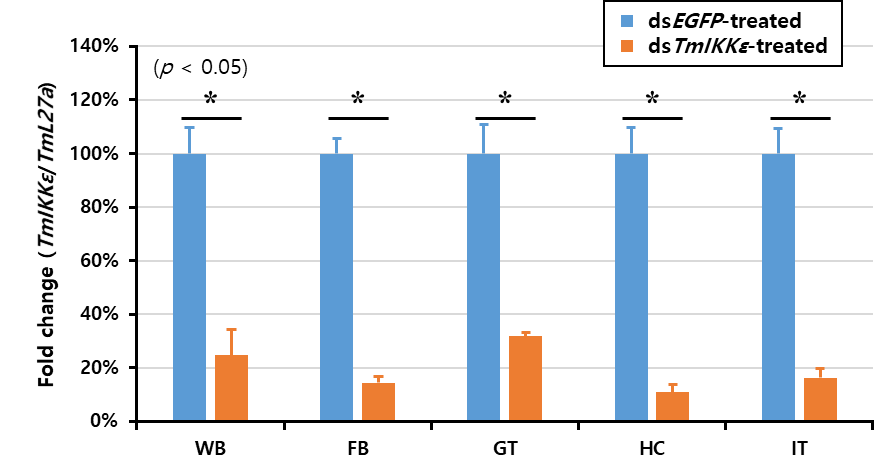


**Supplementary figure 3.** The tissue-specific knockdown efficiency in the *TmIKKε* dsRNA-treated *T. molitor* larvae. The *EGFP* dsRNA-treated larvae served as a negative control. Data represent the mean ± SE of three independent biological replicates.
